# Return‐To‐Work and Working Status After Surgery for Gastric and Esophageal Cancer: A Prospective Observational Study

**DOI:** 10.1002/ags3.70251

**Published:** 2026-07-26

**Authors:** Kentaro Goto, Shigeo Hisamori, Kohei Ueno, Hiroyasu Abe, Ryosuke Okamura, Hisahiro Hosogi, Yoshito Yamashita, Dai Manaka, Hiroaki Hata, Tatsuto Nishigori, Sanae Nakajima, Michihiro Yamamoto, Koichi Kinoshita, Shintaro Okumura, Masazumi Sakaguchi, Shigeru Tsunoda, Yu Sakagami, Koya Hida, Kazutaka Obama

**Affiliations:** ^1^ Department of Surgery Kyoto University Kyoto Japan; ^2^ Department of Regulatory Science and Pharmaceutical Informatics, School of Pharmaceutical Sciences Wakayama Medical University Wakayama Japan; ^3^ Department of Surgery Osaka Red Cross Hospital Osaka Japan; ^4^ Department of Gastrointestinal Surgery Japanese Red Cross Wakayama Medical Center Wakayama Japan; ^5^ Department of Surgery Kyoto‐Katsura Hospital Kyoto Japan; ^6^ Department of Surgery National Hospital Organization Kyoto Medical Center Kyoto Japan; ^7^ Department of Gastrointestinal Surgery Kyoto City Hospital Kyoto Japan; ^8^ Department of Surgery Kobe City Medical Center West Hospital Hyogo Japan; ^9^ Department of Gastrointestinal Surgery Tenri Yorozu Hospital Nara Japan; ^10^ Department of Surgery Japan Baptist Hospital Kyoto Japan; ^11^ Occupational Welfare Division, Agency for Health, Safety and Environment Kyoto University Kyoto Japan

**Keywords:** cancer survivorship, gastroesophageal cancer, health‐related quality of life, patient‐reported outcomes, unemployment

## Abstract

**Aim:**

As survival after gastric and esophageal cancer continues to improve, sustained work participation has become an important survivorship outcome. We aimed to evaluate return‐to‐work (RTW) rates 18 months after curative‐intent surgery for gastric and esophageal cancers and to identify clinical and socioeconomic factors associated with delayed or failed RTW.

**Methods:**

This multicenter, longitudinal, prospective cohort study evaluated working‐age Japanese patients undergoing curative‐intent surgery for gastric or esophageal cancer. Working status was assessed preoperatively and at 6, 12, and 18 months postoperatively. The primary outcome was working status at 18 months. Secondary outcomes included time to first RTW, and factors associated with non‐working and delayed RTW. Exploratory analyses evaluated 6‐month patient‐reported outcomes (QLQ‐C30) and postoperative weight loss.

**Results:**

Among 158 eligible patients, 124 (78.5%) were working at 18 months. Older age (≥ 65 years) and pathological stage≥ III were associated with non‐working at 18 months, whereas sedentary work and self‐employment were associated with a lower risk of non‐working. Postoperative appetite loss, financial difficulties (QLQ‐C30), and ≥ 10% body weight loss at 6 months were associated with non‐working status. Median time to first RTW was 30 and 70 days for gastric and esophageal cancers, respectively. Esophageal cancer and advanced stage were consistently associated with delayed RTW, with weaker evidence for associations with female sex and preoperative retirement.

**Conclusions:**

Approximately 80% of patients were working at 18 months after surgery. Postoperative nutritional impairment and symptom burden were associated with long‐term work participation and may help identify patients at risk of not working after surgery.

## Graphical abstract

In this multicenter prospective study of 158 working‐age patients undergoing curative‐intent surgery for gastric or esophageal cancer, 78.5% were working at 18 months. Postoperative appetite loss, financial difficulties, and ≥ 10% weight loss at 6 months were associated with non‐working status. These findings suggest that postoperative symptoms, nutritional status, and financial vulnerability may help identify patients at risk of not working after surgery.

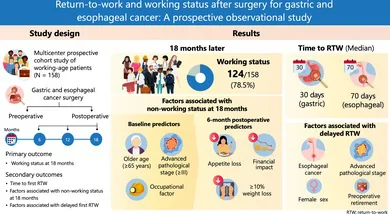

## 1 Introduction

Gastric and esophageal cancers remain major global health burdens [1]. Advances in multimodal treatment strategies, including adjuvant chemotherapy and immunotherapy, have improved long‐term survival in recent years [2, 3, 4]. As survival improves, increasing attention has been directed toward postoperative functional recovery and quality of life (QoL). Together, these domains form part of the broader concept of cancer survivorship, which extends beyond clinical outcomes to include social reintegration.

Return‐to‐work (RTW), a key survivorship indicator, represents an important measure of postoperative recovery and social participation. With prolonged survival and increasing retirement ages in many countries, the number of working‐age cancer survivors is expected to rise, emphasizing the importance of employment maintenance within survivorship care [5]. However, cancer survivors often experience greater difficulty in resuming or maintaining employment than the general population [6, 7]. Poor patient‐reported health status at 6 months after the onset of sick leave was associated with delayed RTW during long‐term follow‐up of up to 18 months in previous studies [8, 9]. Financial toxicity has also been identified as an important concern after cancer surgery [10]. In particular, patients with gastric or esophageal cancer experience postoperative weight loss, nutritional impairment, and decreased activities of daily living following surgery [11, 12, 13], highlighting the need for dedicated RTW support in this population.

Previous cohort‐ and registry‐based studies have reported RTW or employment rates of approximately 70%–90% in gastric cancer survivors and 50%–70% in esophageal cancer survivors [14, 15, 16, 17, 18]. Identified risk factors for delayed or failed RTW include older age, female sex, total gastrectomy, poor performance status, jejunostomy placement, and self‐employment [14, 16, 18]. However, most prior studies were retrospective and often had heterogeneous inclusion criteria and limited follow‐up periods. To our knowledge, no study has prospectively evaluated RTW and associated clinical and socioeconomic factors in a multicenter setting.

To address these gaps, we aimed to evaluate postoperative RTW rates 1.5 years after curative resection for gastric and esophageal cancer. We also aimed to identify the clinical and socioeconomic factors associated with delayed or failed RTW, thereby providing evidence to improve survivorship care for patients with upper gastrointestinal cancer.

## 2 Methods

### 2.1 Patients

This prospective multicenter collaborative study involved eight community hospitals and one university hospital in Japan. Patients aged ≥ 20 years diagnosed with esophageal or gastric cancer with clinical stages I to IVA at the UICC‐TNM criteria, who were employed at the time of diagnosis and were supposed to receive curative surgery from September 2022 to March 2024, were enrolled for this study. After enrolment, patients diagnosed with intraoperative peritoneal dissemination were excluded from follow‐up. Patients who eventually underwent R2 resection or those with a preoperative lack of willingness to RTW were excluded from the analysis. Further eligibility details are provided in the Supplementary Methods.

### 2.2 Perioperative Management

Surgeries and postoperative follow‐up were performed according to relevant Japanese clinical practice guidelines and local institutional practice. Further details are provided in the Supplementary Methods.

### 2.3 Data Collection

After written informed consent was obtained, the participants answered self‐administered paper questionnaires four time points: at enrolment and at regular outpatient visits at 6, 12, and 18 months post‐surgery. The questionnaire administered preoperatively contained inquiries about the patients' social and occupational background. Data on postoperative employment were also collected, including the working status, employment type, and retirement experience until the visit. Additionally, the participants completed the European Organization for Research and Treatment of Cancer (EORTC) QLQ‐C30. Further details of the questionnaire and data management are provided in the Supplementary Methods.

### 2.4 Outcomes

The primary endpoint in this study was the proportion of patients who were working at 18 months post‐surgery. Working status was defined as having worked for at least 1 h during the preceding week at each survey time point. The 18‐month time point was selected to capture sustainable work participation beyond the typical 1‐year period of adjuvant treatment, which is often a major barrier to working resumption. The denominator was set as the full study sample eligible for analysis.

Secondary endpoints were the proportion of employed patients 18 months post‐surgery (including those on sick leave) and time to RTW. Employment status was assessed separately from working status. Time zero was defined as the date of surgery for the main analyses; for sensitivity analyses, time zero was alternatively defined as the start date of treatment or as the last working date.

### 2.5 Predictors

We assessed factors associated with non‐working status at 18 months or delayed RTW. Candidate predictors were selected a priori based on their clinical relevance. To avoid overadjustment, variables considered potential mediators were not included in the primary baseline predictor models. Analyses, including the 6‐month QLQ‐C30 symptoms and body weight loss, were conducted as exploratory and were not incorporated into the primary baseline predictor models to avoid overadjustment. Detailed variable definitions are provided in the Supplementary Methods.

### 2.6 Statistical Analysis

Categorical variables are presented as numbers (percentages), and continuous variables as medians (interquartile ranges) and ranges (minimum–maximum). For the timing of first RTW, the median time and interquartile range (IQR) from surgery to first RTW was estimated using the Kaplan–Meier method.

To assess the factors associated with non‐working at 18 months postoperatively, risk factors were examined using modified Poisson regression with robust standard errors, and risk ratios (RRs) with 95% confidence intervals (CIs) were calculated. Primary risk factor analyses were conducted with patients lost to follow‐up, including those with disease progression or death before 18 months, treated as non‐working at 18 months postoperatively. This conservative approach was used because excluding patients who could not be followed up for 18 months, particularly those with unfavorable clinical courses, could overestimate the proportion of patients working in the full eligible cohort. To explore whether the associations between the main baseline predictors and non‐working status differed by cancer type, interaction terms between cancer type and each predictor were added one at a time to the multivariable modified Poisson model. For the sensitivity analyses, we restricted the dataset to patients who responded to the questionnaire at 18 months to assess robustness to outcome missingness.

To identify the factors associated with the timing of first RTW, we used a competing risks approach. Disease worsening or death that resulted in the discontinuation of follow‐up was defined as a competing event, and other forms of loss to follow‐up were censored. The cumulative incidence of first RTW was estimated using cumulative incidence functions, and factors associated with first RTW were evaluated using Fine–Gray regression to obtain sub‐distribution hazard ratios (SHRs) with 95% CIs.

We used Stata/SE (version 19.0; StataCorp, College Station, TX, USA) for the statistical analysis. The statistical significance threshold was set at a two‐sided *p*‐value of < 0.05. Further details are provided in the Supplementary Methods.

## 3 Results

### 3.1 Patient Characteristics

Figure S1 summarizes the patient flow. Overall, 167 patients were initially enrolled in this study, following the exclusion of 5. Two patients were excluded because they withdrew before postoperative assessment. In addition, patients who lacked willingness to RTW preoperatively (*n* = 1) and those who had missing critical outcome data (*n* = 1) were excluded from the analysis. A total of 158 patients were finally included in the analysis.

Table 1 summarizes the baseline clinicopathological, occupational, and socioeconomic characteristics of the patients. The median age was 65.5 years (range: 37–84 years), and 152 (96.2%) patients achieved R0 resection. Pathological stage≥ III disease was observed in approximately 30% patients. Postoperative complications were observed in 45 (28.5%) patients. Additional perioperative details are summarized in Table S1, which shows that more than 90% of the patients underwent minimally invasive surgery. Preoperative retirement was observed in seven (4.4%) patients, 55 (34.8%) patients were self‐employed, 54 (34.2%) were regular employees, and 96 (60.8%) worked in companies or offices with 50 or fewer employees.

**TABLE 1:** Baseline clinicopathological, occupational, and socioeconomic characteristics.

			Gastric cancer cases (*n* = 111)	Esophageal cancer cases (*n* = 47)	Unavailable data,*n* (%)
Perioperative clinicopathological characteristics					
	Age, years, median (range)		65 (37–82)	67 (42–84)	—
	Sex	Male:Female	78:33	39:8	—
	Body mass index (median [IQR])		23.4 (21.4–25.5)	22.4 (19.9–24.3)	—
	ECOG‐PS	0	107 (96.4%)	40 (85.1%)	—
		1	4 (3.6%)	7 (14.9%)	—
	ASA‐PS	1	20 (18.0%)	2 (4.3%)	—
		2	83 (74.8%)	41 (87.2%)	—
		3	8 (7.2%)	4 (8.5%)	—
	Clinical stage	≥ III	25 (22.5%)	26 (55.3%)	—
	Hypertension		50 (45.0%)	19 (40.4%)	—
	Diabetes mellitus		17 (15.3%)	7 (14.9%)	—
	Ischemic heart disease		7 (6.3%)	2 (4.3%)	—
	Dialysis		0 (0.0%)	1 (2.1%)	—
	Cerebral vascular disease		2 (1.8%)	3 (6.4%)	—
	Smoking history within 1 year		26 (23.4%)	13 (27.7%)	—
	COPD		6 (5.4%)	3 (6.4%)	—
	Preoperative treatment	ESD	11 (9.9%)	2 (4.3%)	—
		Chemotherapy	6 (5.4%)	24 (51.1%)	—
	Histopathology	Adenocarcinoma	111 (100.0%)	9 (19.2%)	—
		Squamous cell carcinoma	0 (0.0%)	38 (80.8%)	—
	Residual tumor	R0	105 (94.6%)	47 (100.0%)	—
		R1	6 (5.4%)	0 (0.0%)	—
	Pathological stage	≥ III	29 (26.1%)	16 (34.0%)	—
	Postoperative complications	All	18 (16.2%)	27 (57.4%)	—
		≥ Grade II	13 (11.7%)	19 (40.4%)	—
	Adjuvant chemotherapy		42 (37.8%)	9 (19.1%)	—
Occupational factors					
	Working hours per week (median [IQR])		40 (24–45)	39 (24–50)	—
	Job resignation soon after cancer diagnosis		6 (5.4%)	1 (2.1%)	—
	Occupation	Sedentary worker	47 (42.3%)	19 (40.4%)	3 (1.9%)
		Non‐sedentary worker	63 (56.8%)	26 (55.3%)	3 (1.9%)
	Employment type	Regular employee	41 (36.9%)	13 (27.7%)	—
		Non‐regular employee	34 (30.6%)	15 (31.9%)	—
		Self‐employed	36 (32.4%)	19 (40.4%)	—
	Employee number at workplace < 50		70 (63.1%)	26 (55.3%)	1 (0.6%)
Socioeconomic factors					
	Education level (University/college)		38 (34.2%)	18 (38.3%)	—
	Head of household		89 (80.2%)	38 (80.9%)	—
	Married		81 (73.0%)	42 (89.4%)	2 (1.3%)
	Monthly personal income < 200 000 JPY		33 (29.7%)	15 (31.9%)	1 (0.6%)
	Monthly household income < 200 000 JPY		18 (16.2%)	7 (14.9%)	1 (0.6%)

*Note:* Values are *n* (%) unless otherwise indicated. Percentages are based on available data for each variable.

Abbreviations: ASA‐PS, American Society of Anesthesiologists physical status; COPD, chronic obstructive pulmonary disease; ECOG‐PS, Eastern Cooperative Oncology Group physical status; ESD, endoscopic submucosal dissection; IQR, interquartile range; JPY, Japanese Yen.

### 3.2 Postoperative Working and Employment Status

The working and employment status, and retirement experience at 6, 12, and 18 months postoperatively are summarized in Table 2. The number of working patients at 18 months post‐surgery was 124 (78.5% [95% CI: 72.0%–85.0%]).

**TABLE 2:** Working and employment status and retirement experience at postoperative months 6, 12, and 18.

	6 months after surgery		12 months after surgery		18 months after surgery	
	Gastric cancer (*n* = 111)	Esophageal cancer (*n* = 47)	Gastric cancer (*n* = 111)	Esophageal cancer (*n* = 47)	Gastric cancer (*n* = 111)	Esophageal cancer (*n* = 47)
Working	89 (80.2%)	34 (72.3%)	87 (78.4%)	38 (80.9%)	90 (81.1%)	34 (72.3%)
Employed	90 (81.1%)	37 (78.7%)	91 (81.9%)	38 (80.9%)	92 (82.9%)	35 (74.5%)
Retirement experience	10 (9.0%)	5 (10.6%)	15^a^ (13.5%)	7 (14.9%)	18^a^ (16.2%)	8 (17.0%)

*Note:* Values are *n* (%) unless otherwise indicated. “Retirement experience” indicates whether the respondent had any retirement experience up to that point.

^a^ Two patients did not answer the question about retirement experience; these were counted as “no retirement experience.”

Employment was maintained by 127 (80.4%) patients at 18 months postoperatively; however, 26 (16.5%) patients had retired by 18 months postoperatively. Moreover, among the 124 working patients at 18 months postoperatively, 8 (6.5%) had retired.

### 3.3 Factors Associated With Non‐Working at 18 Months

Non‐working at 18 months was observed in 34 (21.5%) of the 158 patients, including those who could not be followed up until 18 months.

Table 3 summarizes the univariable and multivariable modified Poisson regression analyses of the clinicopathological and socioeconomic factors associated with non‐working status at 18 months. In multivariable analysis, age ≥ 65 years (RR 2.18; 95% CI 1.14–4.16; *p* = 0.02) and pathological stage≥ III (RR 3.57; 95% CI 1.94–6.57; *p* < 0.001) were associated with non‐working status at 18 months post‐surgery. In contrast, sedentary workers (RR 0.44; 95% CI 0.22–0.86; *p* = 0.02) compared with non‐sedentary workers, and self‐employed (RR 0.40; 95% CI 0.23–0.72; *p* = 0.002) and non‐regular workers (RR 0.38; 95% CI 0.18–0.80; *p* = 0.01) compared with regular workers demonstrated a tendency to work at 18 months after surgery. In exploratory interaction analyses, no statistically significant interaction was observed between cancer type and the main baseline predictors of non‐working status at 18 months (Table S2) [Correction added on 5 August 2026, after first online publication: The citation of Table 2 has been corrected to Table S2]. These findings did not provide clear statistical evidence that the associations between the examined predictors and non‐working status differed by cancer type.

**TABLE 3:** Clinicopathological and socioeconomic factors associated with non‐working at 18 months postoperatively.

		*N*	Non‐working at 18 months (*n* = 34)	Working at 18 months (*n* = 124)	Univariable		Multivariable	
					RR (95% CI)	*p*	RR (95% CI)	*p*
Tumor	Esophageal	47	13 (27.7%)	34 (72.3%)	1.46 (0.80–2.67)	0.22	1.43 (0.78–2.60)	0.25
	Gastric	111	21 (18.9%)	90 (81.1%)	Ref	Ref	Ref	Ref
Sex	Female	41	8 (19.5%)	33 (80.5%)	0.88 (0.43–1.79)	0.72	0.94 (0.42–2.08)	0.87
	Male	117	26 (22.2%)	91 (77.8%)	Ref	Ref	Ref	Ref
Age (years)	≥ 65	87	25 (28.7%)	62 (71.3%)	2.27 (1.13–4.55)	0.02	2.18 (1.14–4.16)	0.02
	< 65	71	9 (12.7%)	62 (87.3%)	Ref	Ref	Ref	Ref
Pathological stage	≥ III	45	21 (46.7%)	24 (53.3%)	4.06 (2.22–7.40)	< 0.001	3.57 (1.94–6.57)	< 0.001
	< III	113	13 (11.5%)	100 (88.5%)	Ref	Ref	Ref	Ref
Preoperative retirement	Yes	7	4 (57.1%)	3 (42.9%)	2.88 (1.40–5.91)	0.004	1.74 (0.74–4.06)	0.20
	No	151	30 (19.9%)	121 (80.1%)	Ref	Ref	Ref	Ref
Occupation^a^	Sedentary	66	8 (12.1%)	58 (87.9%)	0.43 (0.21–0.90)	0.02	0.44 (0.22–0.86)	0.02
	Non‐sedentary	89	25 (28.1%)	64 (71.9%)	Ref	Ref	Ref	Ref
Employment type	Non‐regular employee	49	7 (14.3%)	42 (85.7%)	0.64 (0.27–1.51)	0.31	0.38 (0.18–0.80)	0.01
	Self‐employed	55	15 (27.3%)	40 (72.7%)	1.22 (0.63–2.38)	0.54	0.40 (0.23–0.72)	0.002
	Regular employee	54	12 (22.2%)	42 (77.8%)	Ref	Ref	Ref	Ref

*Note:* Patients with discontinuation of follow‐up within 18 months after surgery were handled as “non‐working.”

Abbreviations: CI, confidence interval; Ref, reference; RR, risk ratio.

^a^ 3 patients with missing occupation data preoperatively.

Table 4 shows the associations between clinically relevant [19] QLQ‐C30 symptom scales at 6 months after surgery and non‐working status at 18 months postoperatively. Pain (RR 2.08; 95% CI 1.04–4.15; *p* = 0.04), appetite loss (RR 2.04; 95% CI 1.10–3.77; *p* = 0.02), and financial impact (RR 2.09; 95% CI 1.09–4.01; *p* = 0.03) were the significant factors associated with non‐working at 18 months.

**TABLE 4:** Associations of symptom scales in QLQ‐C30 (with symptoms) and body weight loss at 6 months after surgery with non‐working status at 18 months postoperatively.

	Symptom/BW loss	*N*	Non‐working at 18 months (*n* = 32)	Working at 18 months (*n* = 124)	RR (95% CI)	*p*
Fatigue	+	57	16 (28.1%)	41 (71.9%)	1.61 (0.84–3.10)	0.15
	—	99	16 (16.2%)	83 (83.8%)	Ref	Ref
Pain	+	29	12 (41.4%)	17 (58.6%)	2.08 (1.04–4.15)	0.04
	—	127	20 (15.8%)	107 (84.3%)	Ref	Ref
Nausea/Vomiting	+	65	16 (24.6%)	49 (75.4%)	1.35 (0.73–2.49)	0.34
	—	91	16 (17.6%)	75 (82.4%)	Ref	Ref
Sleep disturbance	+	17	6 (35.3%)	11 (64.7%)	1.51 (0.73–3.14)	0.27
	—	139	26 (18.7%)	113 (81.3%)	Ref	Ref
Dyspnea	+	77	21 (27.3%)	56 (72.7%)	1.57 (0.76–3.26)	0.23
	—	79	11 (13.9%)	68 (86.1%)	Ref	Ref
Appetite loss	+	36	13 (36.1%)	23 (63.9%)	2.04 (1.10–3.77)	0.02
	—	120	19 (15.8%)	101 (84.2%)	Ref	Ref
Constipation	+	11	4 (36.4%)	7 (63.6%)	1.44 (0.63–3.32)	0.39
	—	145	28 (19.3%)	117 (80.7%)	Ref	Ref
Diarrhea^a^	+	100	21 (21.0%)	79 (79.0%)	1.06 (0.56–2.02)	0.86
	—	55	11 (20.0%)	44 (80.0%)	Ref	Ref
Financial impact^a^	+	60	19 (31.7%)	41 (68.3%)	2.09 (1.09–4.01)	0.03
	—	95	13 (13.7%)	82 (86.3%)	Ref	Ref
BW loss ≥ 10% at 6 months^b^	+	71	21 (29.6%)	50 (70.4%)	2.02 (1.09–3.76)	0.03
	—	81	12 (14.8%)	69 (85.2%)	Ref	Ref

*Note:* Patients who discontinued follow‐up within 18 months after surgery were considered “non‐working.” Additionally, two patients with missing data at six months after surgery were excluded. Each score is adjusted according to the preoperative score of each domain, sex, and age (older than 65 years or not).

Abbreviations: BW, body weight; CI, confidence interval; Ref, reference; RR, risk ratio.

^a^ Missing scores for each domain were excluded from that domain.

^b^ Six patients with missing BW data at 6 months were excluded from the body weight loss analysis.

We also investigated the association between body weight loss at 6 months and non‐working status at 18 months. Six patients had missing data on body weight at 6 months after surgery. Body weight loss ≥ 10% demonstrated a significant association with non‐working status at 18 months (RR 2.02; 95% CI 1.09–3.76; *p* = 0.03).

Sensitivity analyses restricted to patients who completed the 18‐month questionnaire showed largely similar results to the primary analysis. Associations for appetite loss, financial impact, and body weight loss ≥ 10% remained statistically significant, whereas the association with pain was attenuated (Tables S3 and S4) [Correction added on 5 August 2026, after first online publication: The citation of Tables 3 and 4 have been corrected to Tables S3 and S4].

### 3.4 Time to First RTW and Factors Associated With Delayed First RTW

Figure 1 shows the cumulative incidence curve for first RTW stratified by tumor type. The median time to the first RTW was 30 (IQR: 18–69) days for gastric cancer and 70 (IQR: 38–143) days for esophageal cancer. Among the 158 eligible patients, 99 (89.2%) with gastric cancer and 43 (91.5%) with esophageal cancer experienced RTW at least once during follow‐up. Additional cumulative incidence curves stratified by gastrectomy type and for RTW of ≥ 5 h per day are shown in Figures S2 and S3, respectively.

**FIGURE 1:**
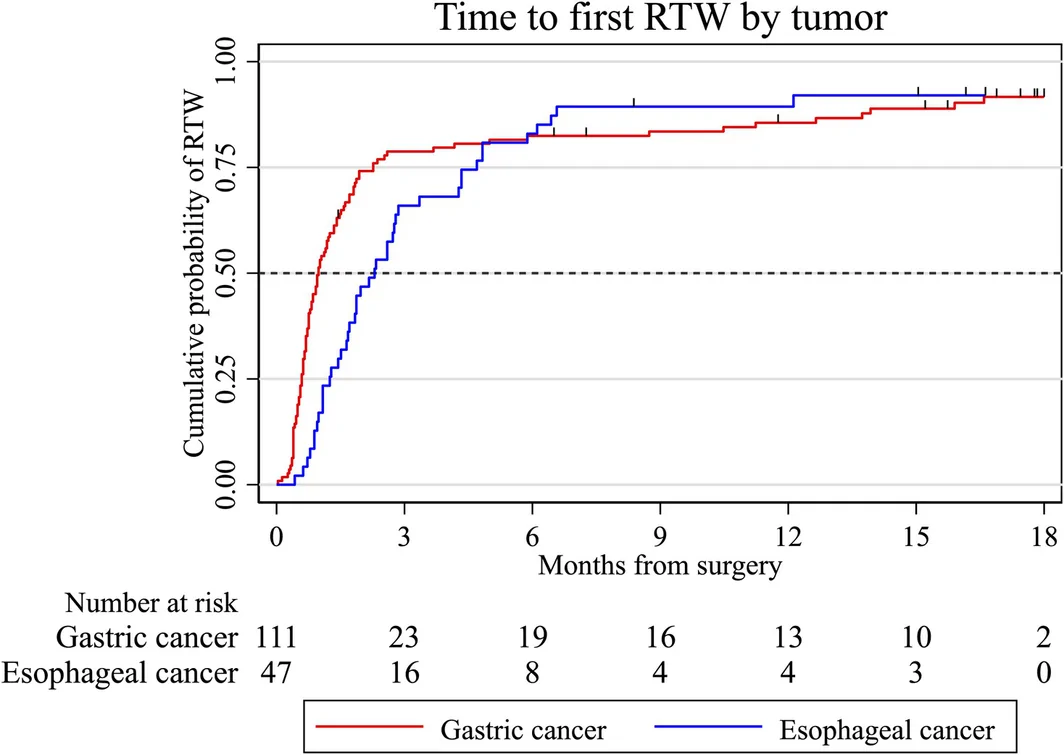
Cumulative first‐RTW curve of the entire cohort stratified by the tumor type. Abbreviation: RTW, return‐to‐work.

Table 5 depicts univariable and multivariable Fine‐Gray analyses of factors associated with delay in the first RTW. In the multivariable analysis, esophageal cancer (SHR 0.59; 95% CI 0.42–0.82; *p* = 0.002), female sex (SHR 0.60; 95% CI 0.38–0.96; *p* = 0.03), pathological stage≥ III (SHR 0.45; 95% CI 0.29–0.70; *p* < 0.001), and preoperative retirement (SHR 0.43; 95% CI 0.20–0.91; *p* = 0.03) were the independent factors associated with delay in the first RTW.

**TABLE 5:** Clinicopathological and socioeconomic factors associated with first‐RTW delay.

		*N*	3 months cumulative RTW	6 months cumulative RTW	Univariable		Multivariable	
					SHR (95% CI)	*p*	SHR (95% CI)	*p*
Tumor	Esophageal cancer	47	66.3%	74.4%	0.71 (0.52–0.96)	0.03	0.59 (0.42–0.82)	0.002
	Gastric cancer	111	78.6%	85.5%	Ref		Ref	
Sex	Female	41	62.4%	71.2%	0.64 (0.43–0.94)	0.02	0.60 (0.38–0.96)	0.03
	Male	117	78.4%	85.7%	Ref		Ref	
Age (years)	≥ 65	87	68.8%	77.1%	0.72 (0.52–0.99)	0.04	0.83 (0.55–1.25)	0.37
	< 65	71	80.4%	87.2%	Ref		Ref	
Pathological stage	≥ III	45	50.2%	59.5%	0.38 (0.26–0.58)	< 0.001	0.45 (0.29–0.70)	< 0.001
	< III	113	83.7%	90.5%	Ref		Ref	
Preoperative retirement	Yes	7	38.2%	45.7%	0.34 (0.13–0.88)	0.03	0.43 (0.20–0.91)	0.03
	No	151	75.8%	83.4%	Ref		Ref	
Occupation^a^	Sedentary worker	66	82.7%	89.4%	1.60 (1.16–2.22)	0.004	1.40 (0.99–1.98)	0.06
	Non‐sedentary worker	89	66.5%	75.3%	Ref		Ref	
Employment type	Non‐regular employee	49	70.9%	79.2%	0.73 (0.50–1.06)	0.10	1.00 (0.65–1.55)	0.99
	Self‐employed	55	68.7%	77.2%	0.68 (0.46–1.02)	0.06	0.90 (0.52–1.56)	0.71
	Regular employee	54	81.7%	88.5%	Ref	Ref	Ref	Ref

Abbreviations: CI, confidence interval; Ref, reference; RTW, return‐to‐work; SHR, sub‐distribution hazard ratio.

^a^ Three patients with missing occupation data preoperatively.

Sensitivity analyses using alternative definitions of time zero showed similar overall findings. Although absolute median times varied, the overall pattern by tumor type was unchanged (Figure S4a,b). Associations for esophageal cancer and advanced pathological stage remained robust, whereas those for female sex and preoperative retirement were less consistent across models (Tables S5 and S6) [Correction added on 5 August 2026, after first online publication: The citation of Table S6 and S7 have been corrected to Table S5 and S6].

## 4 Discussion

In this multi‐institutional prospective cohort of patients undergoing curative‐intent surgery for gastric or esophageal cancer, approximately 80% of respondents were working at 18 months after surgery. Furthermore, the median time to first RTW was 30 days for gastric cancer and 70 days for esophageal cancer. Older age and advanced pathological stage were associated with non‐working at 18 months, whereas appetite loss, financial difficulties, and weight loss at 6 months may serve as practical markers to trigger targeted survivorship and vocational support during routine follow‐up to promote sustainable work participation. Reflecting Japan's aging workforce, our relatively older cohort may increase the relevance of these findings to aging workforces worldwide.

The working proportion at 18 months in our cohort was higher than that reported in several previous studies from other countries (64%–69%) [14, 15, 16, 17, 18]. The time to first RTW was also shorter than that reported in a Japanese study focusing on employees of large corporations [15]. Differences in the study population and occupational context may partly explain these discrepancies. Our cohort included a substantial proportion of self‐employed and sedentary workers, which may have contributed to higher work participation through greater autonomy or lower physical work demands. Additionally, minimally invasive surgery accounted for > 90% of the procedures in our cohort and may reflect contemporary, less invasive surgical practice. However, because no open‐surgery comparison group was available, its contribution to the high RTW rate remains speculative.

Yet, the working proportion in our cohort appears higher than that reported after lung cancer surgery and comparable to that reported in a contemporary colorectal cancer cohort study (68%–79%) [20, 21]. Although direct comparisons across studies should be interpreted with caution, these observations suggest that contemporary gastric and esophageal cancer surgeries may be followed by high work participation and relatively early RTW. Nevertheless, among the 124 working patients at 18 months postoperatively, 8 (6.5%) had experienced retirement, suggesting that some patients left their previous jobs after surgery. This indicates that a non‐negligible subset of patients may return to work yet struggle with sustained job retention and workplace reintegration. In addition, working status should not be equated with full functional recovery or complete workplace reintegration, because patients classified as working may still differ in working hours, work intensity, workplace accommodations, and work performance. Therefore, survivorship care should incorporate vocational support and workplace coordination to prevent avoidable job loss.

Non‐working status at 18 months was associated with oncologic severity, older age, and occupational factors. Advanced pathological stage and age dichotomized at 65 years were associated with the non‐working status at 18 months, which is consistent with prior evidence that older age and more intensive disease or treatment courses are linked to poorer employment outcomes among cancer survivors [22]. The observed disadvantage of non‐sedentary (physically demanding) work is in line with a cross‐cancer systematic review that reported heavy work as a barrier to RTW [23]. In addition, the expansion and persistence of remote or hybrid work after the coronavirus disease pandemic may have further lowered the barriers to work continuation, particularly for sedentary occupations, as work‐from‐home arrangements expanded rapidly in Japan during the pandemic [24]. However, this association may also reflect unmeasured workplace factors, including opportunities for remote or hybrid work, workplace flexibility, and employer accommodations, as well as residual confounding by socioeconomic circumstances. Therefore, the observed association should not be interpreted as evidence that sedentary work itself facilitates work participation. In this occupational context, self‐employment was a protective factor in our cohort, in contrast to the results of a previous European systematic review [22]. This discrepancy may reflect contextual heterogeneity, rather than clinical recovery alone. More than half of our participants worked in small enterprises with limited occupational health support and restricted access to sick leave [25]. In such small companies, continued employment may be more challenging for regular employees, whereas self‐employed individuals may continue working because of greater autonomy in scheduling and flexible task allocation. Alternatively, continued work among self‐employed patients may also reflect financial necessity rather than better clinical recovery, because stopping work can directly lead to income loss in the absence of employer‐based sick‐leave or wage‐replacement benefits. This mechanism is conceptually related to presenteeism, in which patients continue working despite ongoing symptoms or impaired work performance. Therefore, the apparent protective association of self‐employment should not be interpreted solely as evidence of better postoperative recovery. More broadly, associations related to the employment type may be particularly sensitive to contextual factors such as access to sickness benefits and occupational health support. Cross‐study comparisons should be interpreted cautiously because sick pay and wage replacement systems differ markedly across countries. For example, time‐limited statutory sick pay in the United Kingdom, prolonged wage continuation in the Netherlands, and the absence of a universal paid sick‐leave mandate in the United States could influence work participation independent of the postoperative health status in each country [26, 27].

Additionally, 6‐month patient‐reported appetite loss, financial toxicity, and body weight loss were associated with the non‐working status at 18 months, suggesting that mid‐term postoperative recovery profiles may help in identifying patients at risk of long‐term work disability. Previous studies on RTW did not assess these factors, which are specific to the postoperative course of upper gastrointestinal tumors. Appetite loss may contribute to progressive weight loss and reduced physical reserve, thereby compromising work capacity, particularly in patients with a persistent postoperative symptom burden and limited workplace flexibility. Indeed, postoperative weight loss has been investigated primarily as an oncologic prognostic marker [12, 28]. Our findings suggest that postoperative nutritional and symptom assessments may help identify patients at risk of not working. The effectiveness of targeted supportive care in improving sustained work participation requires prospective validation.

Furthermore, in the main Fine–Gray model, esophageal cancer, female sex, advanced pathological stage, and preoperative retirement were independently associated with delayed first RTW; sensitivity analyses using alternative definitions of time zero confirmed the associations for esophageal cancer and advanced pathological stage, whereas those for female sex and preoperative retirement were directionally consistent but less robust. Compared with gastric cancer, esophageal cancer surgery is typically more invasive and often involves a longer length of postoperative hospital stay and greater postoperative complications, which may delay functional recovery and RTW. The association with female sex may partly reflect differences in household economic responsibility, potentially reducing the financial pressure for early RTW among women (Table S7). The association with advanced stage may also be influenced by the downstream treatment burden, such as adjuvant therapy, which we did not model directly to avoid overadjustment. Preoperative retirement was another notable factor associated with delayed RTW, possibly because patients who leave work before treatment may later have reduced access to employment‐related support and benefits [29].

However, this study has some limitations that should be considered. First, generalizability may be constrained by the Japanese healthcare, employment, and social insurance contexts, including policies that support continued employment beyond the typical retirement age [30]; accordingly, our cohort included a relatively high proportion of older working adults compared with many Western studies. Moreover, the substantial representation of self‐employed patients and those working in small enterprises may reflect an important aspect of the Japanese working population undergoing cancer surgery, but it may also limit the applicability of our findings to settings with different access to employer‐based sick leave, wage‐replacement systems, and occupational health support. In addition, although all participants were employed at diagnosis, we could not fully distinguish cancer‐ or treatment‐related inability to work from planned or age‐related retirement among older patients. Accordingly, the association between age ≥ 65 years and non‐working status should not be interpreted as a direct indicator of cancer‐related work disability. Second, the pooled analyses combined patients with gastric and esophageal cancer. Although exploratory interaction analyses did not show statistically significant interactions with cancer type, these analyses were limited by the sample size and the number of outcome events. Therefore, the pooled estimates should be interpreted as average associations across patients with gastric cancer and those with esophageal cancer rather than as cancer‐specific estimates. Third, the observational design precluded causal inference, and unmeasured factors such as workplace accommodation and detailed job demands may have confounded the observed associations. In addition, because post‐RTW work intensity, reduced working hours, detailed workload, and presenteeism were not comprehensively captured, the binary working status outcome may not fully reflect functional recovery or meaningful social reintegration. We also focused on the symptom domains that are plausibly modifiable and may change after surgery, and for which thresholds have been proposed [19]. Finally, in our modeling strategy, we intentionally avoided including candidate variables that could plausibly be intermediate factors in the causal pathway between baseline perioperative characteristics and work outcomes, or socioeconomic variables closely linked to occupation and employment type to reduce the risk of overadjustment and interpretational ambiguity. However, this approach may have limited insight into the pathways linking perioperative factors to work outcomes.

In conclusion, this prospective multi‐institutional study provides contemporary evidence on work participation and RTW timing after gastric and esophageal cancer surgery and identifies clinically and occupationally meaningful factors associated with non‐working status at 18 months and delayed RTW. Integrating standardized patient‐reported outcome assessment and nutritional monitoring into routine postoperative follow‐up may enable the early identification of high‐risk patients; however, whether multidisciplinary survivorship interventions improve health‐related QoL or sustainable work participation requires prospective validation.

## Author Contributions

**Hisahiro Hosogi:** data curation, writing – review and editing. **Kentaro Goto:** conceptualization, data curation, funding acquisition, investigation, methodology, project administration, visualization, writing – original draft, formal analysis. **Hiroaki Hata:** data curation, writing – review and editing. **Dai Manaka:** data curation, writing – review and editing. **Tatsuto Nishigori:** data curation, writing – review and editing. **Michihiro Yamamoto:** data curation, writing – review and editing. **Yoshito Yamashita:** data curation, writing – review and editing. **Koichi Kinoshita:** data curation, writing – review and editing. **Sanae Nakajima:** data curation, writing – review and editing. **Shigeo Hisamori:** conceptualization, funding acquisition, methodology, project administration, supervision, writing – original draft. **Kohei Ueno:** conceptualization, data curation, formal analysis, funding acquisition, methodology, project administration, writing – review and editing, investigation. **Ryosuke Okamura:** conceptualization, funding acquisition, methodology, project administration, supervision, writing – review and editing. **Hiroyasu Abe:** formal analysis, writing – review and editing. **Shintaro Okumura:** data curation, writing – review and editing. **Koya Hida:** supervision, writing – review and editing. **Shigeru Tsunoda:** data curation, writing – review and editing. **Yu Sakagami:** methodology, writing – review and editing. **Masazumi Sakaguchi:** data curation, writing – review and editing. **Kazutaka Obama:** supervision, writing – review and editing.

## Funding

This work was supported by grants from the Mitsubishi Foundation (202230008) and Japan Society for the Promotion of Science (JSPS) KAKENHI Grant‐in‐Aid for JSPS Fellows (25KJ1576).

## Ethics Statement

This study adhered to the principles of the Declaration of Helsinki and was registered with the University Hospital Medical Information Network Clinical Trials Registry (UMIN000048880). All procedures were performed in compliance with relevant laws and institutional guidelines. This study was approved by the Kyoto University Graduate School and Faculty of Medicine Ethics Committee (R3516) and all the participating centers.

## Consent

Written and signed informed consent was obtained from all participants.

## Conflicts of Interest

The authors declare no conflicts of interest.

## Supporting information


**Table S1:** Additional perioperative details.
**Table S2:** Interaction analyses between cancer type and main baseline predictors for non‐working status at 18 months.
**Table S3:** Clinicopathological and socioeconomic factors associated with non‐working at 18 months postoperatively, restricting the dataset to patients who completed the 18‐month questionnaire.
**Table S4:** Associations of symptom scales in QLQ‐C30 “with symptoms” and body weight loss at 6 months after surgery with non‐working status at 18 months postoperatively, restricting the dataset to patients who completed the 18‐month questionnaire.
**Table S5:** Clinicopathological and socioeconomic factors associated with first‐RTW delay, setting time zero as the start date of treatment.
**Table S6:** Clinicopathological and socioeconomic factors associated with first‐RTW delay, setting time zero as the last working date.
**Table S7:** Relationship between sex and household head status.
**Figure S1:** Patient enrolment flowchart of this study.
**Figure S2:** Cumulative first‐RTW curve of the gastric cancer cohort stratified by the operative procedure.
**Figure S3:** Cumulative first‐RTW curve for ≥ 5 h of work per day in the cohort stratified by the tumor type.
**Figure S4:** Sensitivity analysis of the cumulative first‐RTW curve.
**Supplementary Methods.** Additional methodological details.

## Data Availability

The data that support the findings of this study are available on request from the corresponding author. The data are not publicly available due to privacy or ethical restrictions.
