# Supplementary material for: Return‐To‐Work and Working Status After Surgery for Gastric and Esophageal Cancer: A Prospective Observational Study
Source: Ann Gastroenterol Surg. 2026 Jul 26;10(6):1732–42. doi: 10.1002/ags3.70251 (PMC13402095; doi:10.1002/ags3.70251)
Supplement: Supplementary file 1 [file AGS3-10-1732-s001.docx]

Original Article

**Return-to-work and working status after surgery for gastric and esophageal cancer:
A prospective observational study**

Kentaro Goto ^1^, Shigeo Hisamori ^1^, Kohei Ueno, MD ^1^, Hiroyasu Abe ^2^, Ryosuke Okamura ^1^,
Hisahiro Hosogi ^3^, Yoshito Yamashita ^4^, Dai Manaka ^5^, Hiroaki Hata ^6^, Tatsuto Nishigori ^7^,
Sanae Nakajima ^8^, Michihiro Yamamoto ^9^, Koichi Kinoshita ^10^, Shintaro Okumura ^1^,
Masazumi Sakaguchi ^1^, Shigeru Tsunoda ^1^, Yu Sakagami ^11^, Koya Hida ^1^, Kazutaka Obama ^1^

^1^ Department of Surgery, Kyoto University, Kyoto, Japan

^2^ Department of Regulatory Science and Pharmaceutical Informatics, School of Pharmaceutical Sciences, Wakayama Medical University, Wakayama, Japan

^3^ Department of Surgery, Osaka Red Cross Hospital, Osaka, Japan

^4^ Department of Gastrointestinal Surgery, Japanese Red Cross Wakayama Medical Center, Wakayama, Japan

^5^ Department of Surgery, Kyoto-Katsura Hospital, Kyoto, Japan

^6^ Department of Surgery, National Hospital Organization Kyoto Medical Center, Kyoto, Japan

^7^ Department of Gastrointestinal Surgery, Kyoto City Hospital, Kyoto, Japan

^8^ Department of Surgery, Kobe City Medical Center West Hospital, Hyogo, Japan

^9^ Department of Gastrointestinal Surgery, Tenri Yorozu Hospital, Nara, Japan

^10^ Department of Surgery, Japan Baptist Hospital, Kyoto, Japan

^11^ Occupational Welfare Division, Agency for Health, Safety and Environment, Kyoto University, Kyoto, Japan

**Corresponding Author:**

Shigeo Hisamori

E-mail address: hisamori@kuhp.kyoto-u.ac.jp

Address: 54 Shogoin-Kawahara-cho, Sakyo-ku, Kyoto 606-8507, Japan.
ORCID ID: 0000-0003-0163-6978

**Supplementary Materials - Index**

| **Supplementary Methods** | **Page Number** |
| --- | --- |
| Patients | *4* |
| Perioperative management | *4* |
| Data collection | *4–5* |
| Predictors | *5* |
| Statistical analysis | *6* |
| **Supplementary Figures and Tables** |  |
| Supplementary Table 1 | *7–8* |
| Supplementary Table 2 | *9* |
| Supplementary Table 3 | *10–11* |
| Supplementary Table 4 | *12–13* |
| Supplementary Table 5 | *14–15* |
| Supplementary Table 6 | *16–17* |
| Supplementary Table 7 | *18* |
| Supplementary Figure 1 | *19* |
| Supplementary Figure 2 | *20* |
| Supplementary Figure 3 | *21* |
| Supplementary Figure 4a, b | *22* |
| **References** | *23* |

**Supplementary Methods**

Patients

Employment was defined as work conducted for profit or involvement in unpaid labor within a familial enterprise.

Patients were not included if they were scheduled for treatment of another malignancy between esophageal or gastric cancer diagnosis and surgical admission, required emergency surgery, or underwent non-standard procedures, including salvage surgery, bypass surgery, blunt resection of the esophagus, local resection, or two-stage reconstruction.

Esophagogastric junction tumors were categorized based on the 8th edition of the UICC-TNM criteria: when the center of the tumor was located more than 2 cm distal to the esophageal-gastric junction (EGJ), they were classified as gastric cancer, while those within 2 cm of the EGJ were classified as esophageal cancer.

Perioperative management

Surgeries were performed as standardized procedures with lymph dissection in accordance with the Japanese clinical practice guidelines.^1,2^ To enhance inter-institutional consistency, operative principles were regularly discussed among participating centers through the Kyoto Esophageal and Gastric Surgery Study Group, which includes all study institutions. Postoperative follow-up and oncologic surveillance were conducted during routine outpatient care at each participating institution, guided by relevant Japanese guidelines and local practice.

Data collection

The questionnaire administered preoperatively contained inquiries about the patients' social and occupational background, including the occupation type, employment type, business size, resignation, sick leave, household structure, and income.

Completed questionnaires were returned to the study staff at follow-up visits and forwarded by mail to the central data management office, where responses were entered into a secure study database by designated data managers. Individual questionnaire responses were not shared with the treating clinicians during follow-up and were not routinely available for clinical review. The clinical and pathological data were prospectively retrieved from a study electronic data capture system. Each participant was awarded a prepaid gift card valued at 1,000 JPY as an acknowledgement of gratitude for their involvement.

Predictors

Administrative/managerial, professional/engineering, and clerical personnel were classified as "sedentary workers" according to the occupational categories used in a previous report.^3^

Postoperative weight change was calculated as the percent change from the preoperative body weight to 6 months after surgery: 100 × (weight at 6 months − preoperative weight) / preoperative weight. Clinically relevant weight loss was defined as ≥10% decrease based on previous reports.^4,5^

Patient-reported outcomes were assessed using the EORTC QLQ-C30 preoperatively and at 6 months post-surgery. The scores were linearly transformed to 0–100 according to the EORTC scoring manual. We evaluated all the QLQ-C30 symptom scales, including multi-item symptom scales and single-item symptoms. For the main analyses of postoperative symptoms, we used the 6-month scores dichotomized according to previously reported clinically relevant cut-offs.^6^

Statistical analysis

Regarding the sample size calculation, the expected RTW proportion at 18 months post-surgery was assumed to be approximately 65–70%, based on previous studies on gastric and esophageal cancer. To estimate this proportion, with a precision of approximately 15%, the required sample size was calculated to be approximately 126–165 patients.

For the analysis of non-working status at 18 months, modified Poisson regression with robust standard errors was used to directly estimate risk ratios for binary outcomes.^7^

For time to first RTW, we used a competing-risks framework, in which disease worsening or death leading to discontinuation of follow-up was treated as a competing event and other forms of loss to follow-up were censored. Subdistribution hazard ratios were estimated using the Fine–Gray model.^8^

To assess the factors associated with non-working at 18 months postoperatively, sex, age dichotomized at 65 years, and each preoperative score/weight were used as baseline covariates in the corresponding models.

**Supplementary Figures and Tables**

Supplementary Table 1. Additional perioperative details

|  | | **Gastric cancer cases** (n = 111) | **Esophageal cancer cases** (n = 47) |
| --- | --- | --- | --- |
| **Esophagogastric junctional cancer** | | 6 (5.4%) | 8 (17.0%) |
| **Thoracic approach** | MIS | NA | 47 (100.0%) |
| **Abdominal approach** | MIS | 109 (98.2%) | 45 (95.7%) |
|  | Open | 2 (1.8%) | 2 (4.3%) |
| **Operative procedure** | Distal gastrectomy | 81 (73.0%) | NA |
|  | Total gastrectomy | 21 (18.9%) | NA |
|  | Proximal gastrectomy (including lower esophagectomy) | 9 (8.1%) | 3 (6.4%) |
|  | Subtotal esophagectomy | NA | 44 (93.6%) |
| **Reconstruction route of esophageal** | Antethoracic | NA | 1 (2.1%) |
|  | Retrosternal | NA | 20 (42.6%) |
|  | Posterior mediastinal | NA | 26 (55.3%) |
| **Reconstruction procedure** | Billroth I | 48 (43.2%) | NA |
|  | Billroth II | 17 (15.3%) | NA |
|  | Roux-en-Y | 37 (33.3%) | NA |
|  | Esophageal -remnant gastric | 3 (2.7%) | NA |
|  | Esophageal -gastric tube | 2 (1.8%) | 46 (97.9%) |
|  | Double-tract | 4 (3.6%) | 1 (2.1%) |
| **Postoperative hospital stays (days), median (range)** | | 12 (6–59) | 22 (12–65) |

Values are n (%) unless otherwise indicated.

Lymph node dissections were performed according to standardized procedures, except in one case of pancreaticoduodenectomy, in which D1 dissection was performed.

Abbreviations: MIS, minimally invasive surgery; NA, not applicable

Supplementary Table 2. Interaction analyses between cancer type and main baseline predictors for non-working status at 18 months

| **Interaction term** | **Interaction RR (95% CI)** | **p-value** |
| --- | --- | --- |
| Esophageal cancer × female sex | 0.50 (0.12–2.16) | 0.36 |
| Esophageal cancer × age ≥65 years | 0.90 (0.22–3.68) | 0.88 |
| Esophageal cancer × pathological stage ≥III | 0.32 (0.10–1.06) | 0.06 |
| Esophageal cancer × preoperative retirement | 0.92 (0.24–3.50) | 0.91 |
| Esophageal cancer × sedentary work | 1.09 (0.29–4.14) | 0.90 |
| Esophageal cancer × non-regular employment | 1.17 (0.22–6.27) | 0.85 |
| Esophageal cancer × self-employment | 0.44 (0.11–1.77) | 0.25 |

Each model was based on the original multivariable model and included one interaction term at a time.

Abbreviations: CI, confidence interval; RR, risk ratio.

Supplementary Table 3. Clinicopathological and socioeconomic factors associated with non-working at 18 months postoperatively, restricting the dataset to patients who completed the 18-month questionnaire

|  | | **N** | **Non-working at 18 months** (n = 20) | **Working at 18 months** (n = 124) | **Univariable** | | **Multivariable** | |
| --- | --- | --- | --- | --- | --- | --- | --- | --- |
|  |  |  |  |  | RR (95% CI) | p-value | RR (95% CI) | p-value |
| **Tumor** | Esophageal | 40 | 6 (15.0%) | 34 (85.0%) | 1.11 (0.46–2.71) | 0.81 | 1.18 (0.50–2.75) | 0.71 |
|  | Gastric | 104 | 14 (13.5%) | 90 (86.5%) | Ref | Ref | Ref | Ref |
| **Sex** | Female | 38 | 5 (13.2%) | 33 (86.8%) | 0.93 (0.36–2.39) | 0.88 | 0.99 (0.35–2.81) | 0.99 |
|  | Male | 106 | 15 (14.2%) | 91 (85.8%) | Ref | Ref | Ref | Ref |
| **Age (years)** | ≥ 65 | 77 | 15 (19.5%) | 62 (80.5%) | 2.61 (1.00–6.82) | 0.05 | 2.89 (1.27–6.59) | 0.01 |
|  | < 65 | 67 | 5 (7.5%) | 62 (92.5%) | Ref | Ref | Ref | Ref |
| **Pathological stage** | ≥ III | 33 | 9 (27.3%) | 24 (72.7%) | 2.75 (1.24–6.08) | 0.01 | 2.59 (1.14–5.88) | 0.02 |
|  | < III | 111 | 11 (9.9%) | 100 (90.1%) | Ref | Ref | Ref | Ref |
| **Preoperative retirement** | Yes | 6 | 3 (50.0%) | 3 (50.0%) | 4.06 (1.62–10.2) | 0.004 | 2.33 (0.82–6.67) | 0.11 |
|  | No | 138 | 17 (12.3%) | 121 (87.7%) | Ref | Ref | Ref | Ref |
| **Occupation**^†^ | Sedentary | 62 | 4 (6.5%) | 58 (93.5%) | 0.32 (0.11–0.92) | 0.03 | 0.34 (0.14–0.83) | 0.02 |
|  | Non-sedentary | 80 | 16 (20.0%) | 64 (80.0%) | Ref | Ref | Ref | Ref |
| **Employment type** | Non- regular employee | 48 | 6 (12.5%) | 42 (87.5%) | 0.78 (0.29–2.09) | 0.62 | 0.40 (0.16–0.99) | 0.05 |
|  | Self-employed | 46 | 6 (13.0%) | 40 (87.0%) | 0.82 (0.30–2.18) | 0.68 | 0.28 (0.12–0.65) | 0.003 |
|  | Regular employee | 50 | 8 (16.0%) | 42 (84.0%) | Ref | Ref | Ref | Ref |

^†^2 patients with missing occupation data preoperatively.

Abbreviations: CI, confidence interval; Ref, reference; RR, relative risk

Supplementary Table 4. Associations of symptom scales in QLQ-C30 “with symptoms” and body weight loss at 6 months after surgery with non-working status at 18 months postoperatively, restricting the dataset to patients who completed the 18-month questionnaire

|  | **Symptom /BW loss** | **N** | **Non-working at 18 months (n = 20)** | **Working at 18 months (n = 124)** | **RR (95% CI)** | **p-value** |
| --- | --- | --- | --- | --- | --- | --- |
| **Fatigue** | + | 52 | 11(21.2%) | 41(78.8%) | 2.01 (0.84–4.78) | 0.12 |
|  | - | 92 | 9 (9.8%) | 83 (90.2%) | Ref | Ref |
| **Pain** | + | 24 | 7 (29.2%) | 17 (70.8%) | 1.94 (0.81–4.62) | 0.14 |
|  | - | 120 | 13 (10.8%) | 107 (89.2%) | Ref | Ref |
| **Nausea/Vomiting** | + | 59 | 10 (16.9%) | 49 (83.1%) | 1.36 (0.58–3.18) | 0.48 |
|  | - | 85 | 10 (11.8%) | 75 (88.2%) | Ref | Ref |
| **Sleep disturbance** | + | 17 | 6 (35.3%) | 11 (64.7%) | 2.32 (1.00–5.38) | 0.05 |
|  | - | 127 | 14 (11.0%) | 113 (89.0%) | Ref | Ref |
| **Dyspnea** | + | 70 | 14 (20.0%) | 56 (80.0%) | 2.17 (0.83–5.62) | 0.11 |
|  | - | 74 | 6 (8.1%) | 68 (91.9%) | Ref | Ref |
| **Appetite loss** | + | 34 | 11 (32.4%) | 23 (67.6%) | 3.37 (1.53–7.40) | 0.002 |
|  | - | 110 | 9 (8.2%) | 101 (91.8%) | Ref | Ref |
| **Constipation** | + | 10 | 3 (30.0%) | 7 (70.0%) | 2.15 (0.76–6.03) | 0.15 |
|  | - | 134 | 17 (12.7%) | 117 (87.3%) | Ref | Ref |
| **Diarrhea**^†^ | + | 92 | 13 (14.1%) | 79 (85.9%) | 1.01 (0.43–2.37) | 0.98 |
|  | - | 51 | 7 (13.7%) | 44 (86.3%) | Ref | Ref |
| **Financial impact**^†^ | + | 54 | 13 (24.1%) | 41 (75.9%) | 2.55 (1.06–6.12) | 0.04 |
|  | - | 89 | 7 (7.9%) | 82 (92.1%) | Ref | Ref |
| **BW loss ≥ 10% at  6 months‡** | + | 65 | 15 (23.1%) | 50 (76.9%) | 3.21 (1.27–8.15) | 0.01 |
|  | - | 74 | 5 (6.8%) | 69 (93.2%) | Ref | Ref |

^†^Missing scores for each domain were excluded from that domain.

‡5 patients with missing BW data at 6 months were excluded from the body weight loss analysis.

Each score was adjusted according to the preoperative score of each domain, sex, and age (older than 65 years or not).

Abbreviations: BW, body weight; CI, confidence interval; Ref, reference; RR, risk ratio

Supplementary Table 5. Clinicopathological and socioeconomic factors associated with first-RTW delay, setting time zero as the start date of treatment

|  | | **N** | **3 months Cumulative RTW** | **6 months Cumulative RTW** | **Univariable** | | **Multivariable** | |
| --- | --- | --- | --- | --- | --- | --- | --- | --- |
|  |  |  |  |  | SHR (95% CI) | p-value | SHR (95% CI) | p-value |
| **Tumor** | Esophageal cancer | 47 | 51.4% | 69.6% | 0.65 (0.48–0.89) | 0.007 | 0.56 (0.40–0.77) | < 0.001 |
|  | Gastric cancer | 111 | 66.7% | 83.8% | Ref |  | Ref |  |
| **Sex** | Female | 41 | 51.5% | 70.1% | 0.71 (0.47–1.06) | 0.10 | 0.69 (0.42–1.13) | 0.14 |
|  | Male | 117 | 64.0% | 81.8% | Ref |  | Ref |  |
| **Age (years)** | ≥ 65 | 87 | 55.2% | 73.6% | 0.71 (0.51–0.98) | 0.04 | 0.80 (0.53–1.21) | 0.29 |
|  | < 65 | 71 | 67.8% | 84.7% | Ref |  | Ref |  |
| **Pathological stage** | ≥ III | 45 | 37.4% | 55.3% | 0.38 (0.26–0.57) | < 0.001 | 0.43 (0.27–0.68) | < 0.001 |
|  | < III | 113 | 70.6% | 87.8% | Ref |  | Ref |  |
| **Preoperative retirement** | Yes | 7 | 30.0% | 44.6% | 0.37 (0.13–1.03) | 0.06 | 0.47 (0.19–1.15) | 0.10 |
|  | No | 151 | 62.3% | 80.2% | Ref |  | Ref |  |
| **Occupation**^†^ | Sedentary worker | 66 | 70.9% | 86.6% | 1.59 (1.15–2.20) | 0.005 | 1.44 (0.99–2.10) | 0.06 |
|  | Non-sedentary worker | 89 | 54.0% | 71.8% | Ref |  | Ref |  |
| **Employment type** | Non-regular employee | 49 | 59.4% | 77.7% | 0.79 (0.53–1.16) | 0.22 | 1.19 (0.76–1.87) | 0.45 |
|  | Self-employed | 55 | 54.3% | 72.9% | 0.68 (0.46–1.01) | 0.06 | 1.04 (0.59–1.83) | 0.90 |
|  | Regular employee | 54 | 68.2% | 85.2% | Ref | Ref | Ref | Ref |

^†^3 patients with missing occupation data preoperatively.

Abbreviations: CI, confidence interval; Ref, reference; RTW, return-to-work; SHR, sub- distribution hazard ratio

Supplementary Table 6. Clinicopathological and socioeconomic factors associated with first-RTW delay, setting time zero as the last working date

|  | | **N** | **3 months Cumulative RTW** | **6 months Cumulative RTW** | **Univariable** | | **Multivariable** | |
| --- | --- | --- | --- | --- | --- | --- | --- | --- |
|  |  |  |  |  | SHR (95% CI) | p-value | SHR (95% CI) | p-value |
| **Tumor** | Esophageal cancer | 46 | 54.8% | 66.7% | 0.64 (0.47–0.86) | 0.004 | 0.51 (0.36–0.71) | < 0.001 |
|  | Gastric cancer | 111 | 71.3% | 82.2% | Ref |  | Ref |  |
| **Sex** | Female | 41 | 54.4% | 66.5% | 0.67 (0.45–1.00) | 0.05 | 0.64 (0.39–1.03) | 0.07 |
|  | Male | 116 | 68.8% | 80.3% | Ref |  | Ref |  |
| **Age (years)** | ≥ 65 | 86 | 59.1% | 71.0% | 0.69 (0.50–0.96) | 0.03 | 0.78 (0.51–1.18) | 0.23 |
|  | < 65 | 71 | 72.6% | 83.3% | Ref |  | Ref |  |
| **Pathological stage** | ≥ III | 45 | 41.4% | 53.2% | 0.39 (0.26–0.58) | < 0.001 | 0.44 (0.27–0.70) | < 0.001 |
|  | < III | 112 | 74.8% | 85.9% | Ref |  | Ref |  |
| **Preoperative retirement** | Yes | 7 | 29.3% | 38.3% | 0.31 (0.13–0.76) | 0.01 | 0.34 (0.18–0.66) | 0.001 |
|  | No | 150 | 67.0% | 78.6% | Ref |  | Ref |  |
| **Occupation**^†^ | Sedentary worker | 66 | 72.7% | 83.9% | 1.52 (1.10–2.10) | 0.01 | 1.21 (0.84–1.74) | 0.30 |
|  | Non-sedentary worker | 88 | 57.6% | 70.0% | Ref |  | Ref |  |
| **Employment type** | Non-regular employee | 49 | 60.9% | 73.0% | 0.70 (0.48–1.02) | 0.06 | 0.88 (0.57–1.36) | 0.57 |
|  | Self-employed | 54 | 59.3% | 71.4% | 0.67 (0.45–1.00) | 0.05 | 0.87 (0.50–1.51) | 0.62 |
|  | Regular employee | 54 | 73.9% | 84.6% | Ref | Ref | Ref | Ref |

1 patient with missing data on the last working date.

^†^3 patients with missing occupation data preoperatively.

Abbreviations: CI, confidence interval; Ref, reference; RTW, return-to-work; SHR, sub- distribution hazard ratio

Supplementary Table 7. Relationship between sex and household head status

|  | **Being a household head** | | **Total** |
| --- | --- | --- | --- |
|  | **Yes** | **No** |  |
| **Male** | 110  (94.0%) | 7  (6.0%) | 117 |
| **Female** | 17  (41.5%) | 24  (58.5%) | 41 |
| **Total** | 127 | 31 | 158 |

Being a household head was asked preoperatively.

() shows the proportion of each cell divided by the total number of each sex.

Supplementary Figure 1: Patient enrolment flowchart of this study.

Abbreviation: RTW, return to work.

Supplementary Figure 2. Cumulative first-RTW curve of the gastric cancer cohort stratified by the operative procedure.

Abbreviation: RTW, return to work.

Supplementary Figure 3. Cumulative first-RTW curve for ≥ 5 hours of work per day in the cohort stratified by the tumor type.

Abbreviation: RTW, return to work.

Supplementary Figure 4. Sensitivity analysis of the cumulative first-RTW curve. Figure 4a shows the curve in which time zero was defined as the start date of treatment, including neoadjuvant therapy and ESD, while Figure 4b shows the curve in which time zero was alternatively defined as the last job date.

Abbreviations: ESD, endoscopic submucosal dissection; RTW, return to work.

a.

b.

**References**

1. Kitagawa Y, Ishihara R, Ishikawa H, et al. Esophageal cancer practice guidelines 2022 edited by the Japan Esophageal Society: part 2. Esophagus. 2023; 20(3): 373–89.

2. Japanese Gastric Cancer Association. Japanese Gastric Cancer Treatment Guidelines 2021 (6th edition). *Gastric Cancer*. 2023; 26: 1–25.

3. Choi KS, Kim EJ, Lim JH et al. Job loss and reemployment after a cancer diagnosis in Koreans – A prospective cohort study. Psychooncol*.* 2007; 16(3): 205–13.

4. Climent M, Munarriz M, Blazeby JM et al. Weight loss and quality of life in patients surviving 2 years after gastric cancer resection. Eur J Surg Oncol. 2017; 43(7): 1337–43.

5. Cederholm T, Jensen GL, Correia MITD, et al. GLIM criteria for the diagnosis of malnutrition – a consensus report from the global clinical nutrition community. Clin Nutr. 2019; 38(1): 1–9.

6. Giesinger JM, Loth FLC, Aaronson NK et al. Thresholds for clinical importance were established to improve interpretation of the EORTC QLQ-C30 in clinical practice and research. J Clin Epidemiol*.* 2020; 118: 1–8.

7. Zou G. A modified poisson regression approach to prospective studies with binary data. Am J Epidemiol. 2004; 159(7): 702–6.

8. Fine JP, Gray RJ. A proportional hazards model for the subdistribution of a competing risk. J Am Stat Assoc. 1999; 94(446): 496–509.
